# Cardiometabolic Impact of Encapsulated Cocoa Powder and Pure Cocoa Ingredients Supplementation: A Comparative Placebo‐Controlled RCT in Adults

**DOI:** 10.1002/mnfr.202400490

**Published:** 2025-02-03

**Authors:** Janina Weigant, Anuschka Afchar, Meike Barzen, Lisa Dicks, Benno F. Zimmermann, Matthias Schmid, Leonie Weinhold, Birgit Stoffel‐Wagner, Jörg Ellinger, Peter Stehle, Sabine Ellinger

**Affiliations:** ^1^ Faculty of Agricultural, Nutritional and Engineering Sciences, Institute of Nutritional and Food Science, Human Nutrition University of Bonn Bonn Germany; ^2^ Department of Nutritional and Food Sciences Niederrhein University of Applied Sciences Mönchengladbach Germany; ^3^ Faculty of Agricultural, Nutritional and Engineering Sciences, Institute of Nutritional and Food Science, Food Sciences University of Bonn Bonn Germany; ^4^ Faculty of Medicine, Institute of Medical Biometry, Informatics and Epidemiology (IMBIE) University of Bonn Bonn Germany; ^5^ Faculty of Medicine, Institute of Clinical Chemistry and Clinical Pharmacology University Hospital Bonn Bonn Germany; ^6^ Faculty of Medicine, Department of Urology and Pediatric Urology University Hospital Bonn University Bonn Bonn Germany; ^7^ Faculty of Agricultural, Nutritional and Engineering Sciences, Institute of Nutritional and Food Science, Nutritional Physiology University of Bonn Bonn Germany

**Keywords:** cardiometabolic risk factors, epicatechin, flavanol‐rich cocoa, methylxanthines, pulse wave velocity

## Abstract

Consuming cocoa rich in flavan‐3‐ols (particularly epicatechin [EC]) may reduce vascular stiffness and blood pressure (BP) and improve serum lipid profiles. Because interventional studies on pure EC exhibited inconclusive results, the role of other cocoa ingredients such as methylxanthines (MX) on vascular health was assumed. This study aimed to systematically compare the effects of flavanol‐rich cocoa and its major components EC and MX on vascular function and serum lipid levels. In a randomized controlled trial (RCT), 75 healthy young adults ingested capsules containing either (i) flavanol‐rich cocoa powder, (ii) EC, (iii) MX, (iv) EC + MX, or (v) placebo (*n *= 15 per group) daily for 4 weeks. Capsules provided equal amounts of EC and/or MX as the cocoa capsules. Pulse wave velocity (PWV), BP, endothelin‐1, and lipids were investigated before and after intervention. No group‐specific statistically significant differences in aortic PWV (*p *= 0.410) or any other parameters (*p *≥ 0.05) were observed between before and after the intervention. Daily intake of neither flavanol‐rich cocoa nor pure cocoa ingredients influenced vascular function and lipid profiles in healthy adults. Consequently, RCTs involving subjects with increased cardiometabolic risk may clarify the effects of EC and MX as cocoa components on cardiovascular health parameters.

**Trial Registration**: URL: https://drks.de/search/en/trial. Unique identifier: DRKS00022056

## Graphical abstract

This study compared the impact of cocoa (ingredients) on cardiometabolic health. Healthy young adults ingested capsules with either flavanol‐rich cocoa, epicatechin (EC), methylxanthines (MX), EC + MX, or placebo capsules daily for 4 weeks. Before and afterwards, parameters on vascular function and blood lipids were assessed, but the changes between these treatments were not different. Thus, further investigations should be done in subjects with an already increased cardiometabolic risk.

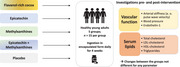

**Abbreviations**
ABI: ankle brachial index
AIX: augmentation index
AIX 75: augmentation index standardized to a heartbeat of 75 beats per minute
BP: blood pressure
CF: cocoa flavan‐3‐ols
DBP: diastolic blood pressure
DP: degree of polymerization
EC: epicatechin
ET‐1: endothelin‐1
FMD: flow‐mediated dilatation
HDL‐C: HDL‐cholesterol
LDL‐C: LDL‐cholesterol
MX: methylxanthines
PRT: radial pulse raise time
PWV: pulse wave velocity
RCT: randomized controlled trial(s)
SBP: systolic blood pressure
TB: theobromine
TC: total cholesterol
TG: triglycerides

## 1 Introduction

Various meta‐analyses of randomized controlled trials (RCTs) have suggested that regular consumption and single ingestion of flavanol‐rich cocoa drinks, chocolate bars, or cocoa powder decrease systolic blood pressure (SBP) [1], diastolic blood pressure (DBP) [1], and pulse wave velocity (PWV) [2] not only in patients with diabetes mellitus, hypertension, or cardiovascular diseases but also in obviously healthy adults belonging to different age groups. Beyond these clinically relevant effects, the consumption of flavanol‐rich cocoa products seems to favorably modify serum lipid profiles; for example, it decreases low‐density lipoprotein‐cholesterol (LDL‐C) and triglycerides (TG) [1, 3, 4, 5] and increases high‐density lipoprotein (HDL)‐cholesterol (HDL‐C) [1, 4, 6]. Thus, the intake of cocoa products can help maintain long‐term vascular health.

To date, only a few studies have been conducted to reveal the mechanism(s) underlying the beneficial cardiometabolic effects observed after cocoa consumption. In a 1‐week interventional study, healthy young adults consumed cocoa drinks daily containing various amounts of a mixture of mono‐ to decameric flavan‐3‐ols (sum of cocoa flavan‐3‐ols [CF] with a degree of polymerization [DP] 1–10: 0–800 mg); the drinks contained matched amounts of theobromine (TB) (329 mg), caffeine (25 mg), and minerals (Na, K, and Mg). Compared with the control (CF‐free drink), 7‐day supplementation with at least 200 mg CF reduced the PWV, SBP, and endothelin‐1 (ET‐1) levels while increasing flow‐mediated dilatation (FMD). However, a higher daily CF intake of up to 500 mg or even 800 mg did not further alter the effects on the PWV, SBP, ET‐1, and FMD; the decrease in DBP not observed with lower CF doses reached significance [7]. A longer‐term RCT involving healthy adults (4 weeks) assessed the effects of daily supplementation with encapsulated cocoa extract containing various quantities and qualities of CF (intervention groups: DP1‐10 containing 130 mg monomeric epicatechin [EC] and 560 mg procyanidins or DP2‐10 containing 20 mg EC and 540 mg procyanidins; control: CF‐free), but similar quantities of TB and caffeine on selected cardiometabolic variables. Compared with the control treatment, only DP1‐10 beneficially modulated the PWV, SBP, and FMD; both DP1‐10 and DP2‐10 decreased the total plasma cholesterol levels compared with the control. Most interestingly, the positive effects observed only with DP1‐10 could be linked to increased plasma levels of EC metabolites [8], further supporting the evidence that the monomer EC is (partly) responsible for the vascular effects of cocoa, probably because of its higher bioavailability than catechin [9, 10, 11] and dimer procyanidin B2 [11, 12]. In subsequent placebo‐controlled RCTs, daily intake of pure encapsulated EC (25 mg per day for 2 weeks [13] or 100 mg per day for 4 weeks [14]) did not have any effect on selected vascular parameters or serum lipids. Based on their results, Sansone et al. [15] assumed that the bioavailability rate of monomeric EC and its metabolic impact are influenced by methylxanthines (MX): bolus ingestion of CF along with theobromine (111 mg) and caffeine (11 mg) augmented the effects of CF on the PWV, FMD, and DBP in healthy adults; ingestion of MX alone did not modulate these parameters. Daily intake of pure TB (500 mg per day [16]; 850 mg per day [17]) for 4 weeks increased fasting HDL‐C levels in healthy adults compared with placebo. However, ingestion of 850 mg TB along with 6 g cocoa had no effects on lipid metabolism parameters in healthy adults [17, 18].

Objectively, the cocoa components that may be responsible for the beneficial cardiometabolic effects of cocoa‐containing products observed in some human intervention trials remain unclear. The aim of the present RCT involving healthy adults was, thus, to systematically compare the impact of cocoa powder and its major components EC and MX on aortic PWV (primary outcome), further vascular parameters and serum lipid levels (secondary outcomes).

## 2 Experimental Section

### 2.1 Participants

Participants were recruited through postings and local public announcements (posters, flyers, and newspaper) in the city of Mönchengladbach and, after the relocation of our working group at the University of Bonn, in the city of Bonn. The inclusion criteria were as follows: healthy nonsmokers aged at least 18 years with a BMI ranging from 18.5 to 29.9 kg m^−2^. The exclusion criteria (only checked using questionnaires due to COVID‐19 pandemic‐related restrictions) were as follows: hypertension (SBP ≥ 140 mm Hg and/or DBP ≥ 90 mm Hg), hypercholesterolemia (total cholesterol [TC] > 200 mg dL^−1^), hypertriglyceridemia (TG > 150 mg dL^−1^), diabetes mellitus, diseases of heart, liver, kidneys, and diseases of the gastrointestinal tract associated with diarrhea or malabsorption, regular medication (including antibiotics), and dietary supplements (including pro‐/prebiotics) that may affect vascular function, including blood pressure (BP) and serum lipids, pregnancy (premenopausal women: urine‐based test at recruitment), lactation, contraindications against pulse wave frequency analysis, and consumption of chocolate (> 100 g per day), red wine (> 1 glass per day), cocoa drink, green or black tea (> 2 cups per day). Written informed consent was obtained from all participants before their inclusion. The study was approved by the Ethics Committee of the University of Bonn (project ID: 145/20; date of approval: 22/04/2020) and was conducted in accordance with the Declaration of Helsinki. The trial was registered in the German Clinical Trials Register (DRKS‐ID: DRK00022056) on June 26, 2020.

### 2.2 Study Design

The trial was conducted as a randomized, placebo‐controlled, double‐blind study with a parallel group design. In total, 75 participants were enrolled in the study by the medical advisor. The subjects were consecutively allocated to five different groups (A, B, C, D, and E) using permuted block randomization (block size of five with five groups per block; allocation ratio 1:1:1:1:1) using sealed envelope software (Sealed Envelope, London, UK) by an individual outside the research staff and saved as an Excel file. The assignment of each participant to the corresponding group was displayed using the filter function in Excel.

Each group received capsules providing either flavanol‐rich cocoa, EC, MX, EC + MX, or maltitol (placebo). The nontransparent capsules (blinding of the participants) for the different treatments were encoded (A, B, C, D, and E) and filled by a pharmacist (Schloss‐Apotheke, Koblenz, Germany) who was not personally involved in the study. The assignment list was stored in a sealed opaque envelope until completion of the statistical evaluation to ensure blinding of the research staff. Capsules were provided in tins with a filter paper on the bottom flavored with a cocoa extract (Dragonspice Naturwaren, Reutlingen, Germany) to provide a uniform smell when opening the tins. The participants were instructed to ingest the capsules twice daily for 4 weeks along with water outside of meals.

Before and after the 4‐week intervention period, vascular stiffness and BP were measured after a 12 h overnight fasting, and anthropometric parameters were investigated. Furthermore, venous blood was collected using Monovettes (Sarstedt, Nümbrecht, Germany) without anticoagulants.

The subjects were instructed to generally maintain their habitual food intake. Three days before the first investigation (run‐in period), the intake of flavan‐3‐ols by food was reduced until study completion by restricting the consumption of flavanol‐rich foods that belong to the major quantitative flavan‐3‐ol sources in Germany [19] (red wine ≤ 1 glass per week, green or black tea ≤ 2 cups per day, fruit juices ≤ 1 glass per day, and apples or pears ≤ 2 pieces per week) and the intake of MX (≤ 4 cups or glasses of caffeine‐containing drinks per day). Cocoa and cocoa‐containing products and energy drinks were avoided during the run‐in period and throughout the intervention. One day before the investigations, the participants were instructed to completely abstain from these products and other alcoholic beverages. Usual physical activities should be maintained throughout the study period.

### 2.3 Capsule Ingredients and Dosage Information

Capsules for the cocoa treatment were filled with 2.5 g ACTICOA cocoa powder, a product complying with the European Food Safety Authority health claim “cocoa flavanols help maintain the elasticity of blood vessels, which contributes to normal blood flow” and providing 200 mg total CF (DP 1–10) according to the manufacturer's instructions (Barry Callebaut, Zurich, Switzerland, lot no. 100‐F017906‐AC‐722). This cocoa powder was enriched with pure TB and caffeine to achieve 329 mg TB and 25 mg caffeine per daily dose (for details, see Table S1) as provided by Grassi et al. [7]. The other test capsules were filled with either pure EC (food grade; purity ≥ 94.9%; N&R Industries, Xi'an, China), pure MX, or a mixture of MX and EC. The EC and MX dosages were matched with the daily EC and MX intake from cocoa treatment (EC, 37.3 mg; TB, 329 mg; caffeine, 25 mg). A powdery mixture of maltitol (99%, wt/wt) and Aerosil (1%, wt/wt; colloidal SiO_2_) was used as a carrier component for the EC/MX capsules and as a filler for the placebo capsules (Table S2).

The content of EC, TB, and caffeine in ACTICOA cocoa powder and the EC preparation were determined using HPLC with UV detection. EC was analyzed as described by Damm et al. [20]. TB and caffeine were determined following method L‐45.001 of the Official Collection of Methods of Analysis (ASU) according to § 64 German Food and Feed Code (LFGB) [21]. Each analysis was performed in duplicate to calculate the mean values.

### 2.4 Vascular Stiffness

Pulse wave frequency analysis was performed to evaluate the effects of cocoa (ingredients) intervention on vascular stiffness, measured by the PWV under standardized conditions as recommended by international organizations [22, 23]. In brief, BP cuffs were applied bilaterally to the upper arm and ankle and to the upper arm and wrist to determine the vascular stiffness of peripheral and aortic vessels, respectively, using VascAssist 2 (inmediQ, formerly iSYMED, Butzbach, Germany). A pressure of 200 mm Hg was generated; after releasing the pressure, the SBP of the brachial and tibial arteries (peripheral measurement) and the brachial and radial arteries (central measurement) was determined. These were applied to calculate parameters of arterial stiffness including aortic PWV as the primary outcome using VascViewer 2 software (inmediQ). Further parameters such as aortic SBP and DBP, compliance index, radial pulse raise time (PRT), resistance index, augmentation index (AIX), and augmentation index standardized to 75 beats per minute (AIX 75), and right and left brachial PWV and right and left ankle brachial index (ABI) were the secondary outcomes. Except for PRT, these parameters were calculated based on a multicompartment model of the arterial system, which has shown to provide realistic pulse waveforms and BP [24, 25]. This device was validated by Massmann et al. [26] and Trinkmann et al. [27].

### 2.5 Office Blood Pressure

SBP and DBP (secondary outcomes) were determined according to the recommendations of the European Society of Hypertension/European Society of Cardiology guidelines [28] using a fully automatic BP monitor (boso carat professional, Bosch & Sohn, Jungingen, Germany).

### 2.6 ET‐1

For ET‐1 analysis, serum samples were immediately frozen at −80°C and stored until study termination. ET‐1 (secondary outcome) was determined at the Institute of Nutritional and Food Science, Human Nutrition, using an ELISA test kit (Quantikine Endothelin‐1 Immunoassay, R&D Systems, Minneapolis, MN, USA; CV 4.0% according to the manufacturer's instructions).

### 2.7 Serum Lipids

TC, LDL‐C, HDL‐C, and TG levels were analyzed using fully automated photometric methods in fresh serum samples on a cobas c702 analyzer (Roche Diagnostics, Mannheim, Germany) according to the manufacturer's instructions at the Institute of Clinical Chemistry and Clinical Pharmacology, University Hospital Bonn. The inter‐assay CV was 1.12% for TC, 0.89% for LDL‐C, 1.08% for HDL‐C, and 1.37% for TG.

### 2.8 Anthropometric Measurements

Body weight and height were measured at the study center, and BMI was calculated accordingly. Waist and hip circumferences were measured according to the WHO recommendations [29], and the waist‐to‐hip ratio was calculated as a parameter of body fat distribution. Fat mass (FM) was determined by bioelectric impedance analysis following the European Society for Clinical Nutrition and Metabolism guidelines [30, 31]. Resistance and reactance were measured at 800 µA and 50 kHz using the BIA 2000–1 device (Data Input, Pöcking, Germany). FM was calculated using the equation of Kyle et al. [32].

### 2.9 Food Intake and Physical Activity

The participants were instructed to document their food intake using standardized 3‐day dietary records before each visit. The mean daily intake of energy and selected nutrients was calculated using the software DGExpert (version 1.9.8.1; German Nutrition Society, Bonn, Germany) based on the official German nutrition database “Bundeslebensmittelschlüssel” (version 3.02), the mean daily intake of EC and total flavan‐3‐ols (sum of (−)‐epicatechin, (+)‐catechin, and procyanidins including di‐ to decamers) using the USDA Database for the Flavonoid Content of Selected Foods (release 3.3) [33] and the USDA Database for the Proanthocyanidin Content of Selected Foods (release 2) [34], and the intake of TB and caffeine applying the USDA Food and Nutrient Database for Dietary Studies (FNDDS) 2021–2023 [35]. If the USDA databases provided several data on the content of (−)‐epicatechin, (+)‐catechin, procyanidins, TB, and caffeine for the same food, the median content was calculated and used to determine the individual intake of these nutrients.

Physical activity (number of steps per day) was assessed for 3 days before each visit using a pedometer (Yamax Digi—Walker CW 700, Bridgnorth, Shropshire, UK).

### 2.10 Compliance

The participants were instructed to record their capsule intake daily in a study diary. Furthermore, the tins with the remaining capsules were returned after completion of the study to calculate compliance: rates ≥ 85% were accepted as proper completion of the study protocol.

### 2.11 Sample Size Estimation

The sample size was estimated based on the PWV (primary outcome), which has been shown to decrease on average by 1.0 m s^−1^ in healthy young adults by daily intake of flavanol‐rich cocoa extract versus flavanol‐free placebo for 1 month [8]. A similar decrease in PWV was expected by daily intake of CF‐rich cocoa powder versus placebo for 4 weeks. Thus, according to the equation of Ott [36], a decrease in PWV of at least 1.0 m s^−1^ with a SD of 1.41 (derived from 95% confidence interval [CI] of Rodriguez‐Mateos et al. [8]) by cocoa compared with placebo treatment can be detected with 13 subjects per group if an *α* of 0.05 and a power of 80% were assumed. Because 15 subjects per group would be required in case of 15% dropout, 75 participants were included in the study.

### 2.12 Statistical Analysis

Participant characteristics are presented as absolute and relative frequencies for categorical variables and as means ± SEM for continuous variables. For group comparisons of continuous variables, the variables were investigated for normal distribution using the Shapiro–Wilk test. If the hypothesis of normality was rejected, the data were logarithmized, provided that the transformed data approximated a normal distribution.

Changes in vascular parameters and serum lipid levels between before and after treatment were compared between the groups using one‐way analysis of variance (one‐way ANOVA) or the Kruskal–Wallis test. Data are presented as means ± SEM if not indicated otherwise. *p* values < 0.05 were considered statistically significant. All statistical analyses were performed using IBM SPSS Statistics (version 27.0; IBM Deutschland, Ehningen, Germany).

## 3 Results

### 3.1 Study Flow

**FIGURE 1:**
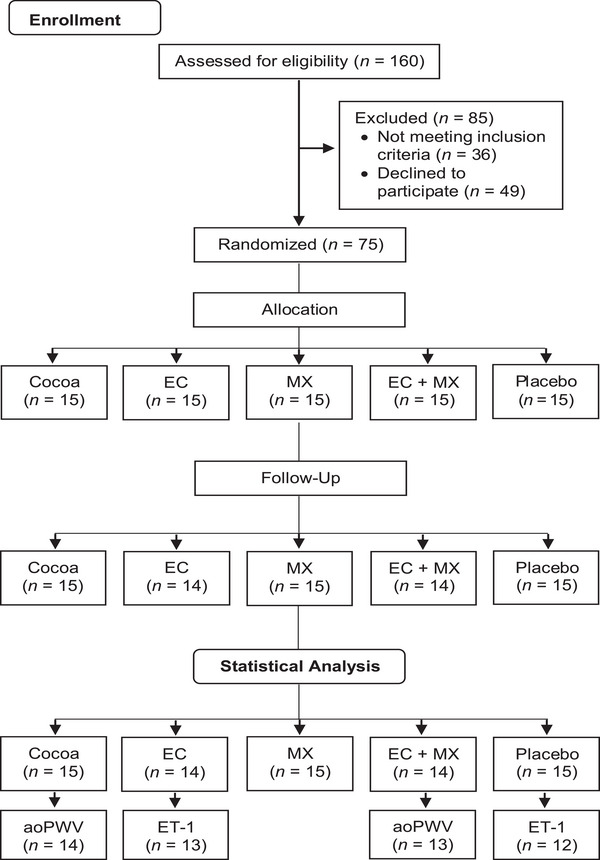
Study flow according to Consolidated Standards of Reporting Trials 2010. Aortic PWV and derived parameters (aortic SBP, aortic DBP, compliance index, pulse raise time, resistance index, augmentation index, augmentation index standardized for 75 beats per minute), and ET‐1 were evaluated based on a reduced number of participants. AoPWV indicates aortic pulse wave velocity; EC, epicatechin; ET‐1, endothelin‐1; MX, methylxanthines.

Seventy‐five participants (15 in each group) started the trial as planned. Two participants (one in the EC group and one in the EC + MX group) dropped out because of personal reasons. Thus, 73 subjects completed the study according to the protocol and were included in the statistical analysis. Due to technical problems during pulse wave measurements, data on aortic PWV and derived parameters (aortic SBP, aortic DBP, compliance index, PRT, resistance index, AIX, and AIX 75) were only available for 71 participants (at least 13 participants per group). ET‐1 analyses could only be performed in 69 subjects due to incomplete blood sampling; therefore, data were only available for 69 individuals (for details, see Figure 1). The calculated mean compliance rate of capsule intake was 99.1% (minimum 89.3%).

### 3.2 Characteristics of the Study Population

Table 1 presents the characteristics of the study population at baseline. Age, sex, and BMI were comparable between the groups. All measurements and analyses were within the reference range for healthy individuals. Anthropometric measurements, dietary intake, and physical activity remained unchanged throughout the intervention period (Table S3). According to the dietary records, all participants followed the food restrictions as requested; therefore, the intake of test substrates by food revealed only a minor share of total intake during the intervention, except for caffeine (Table S3). The adverse effects associated with the intervention were not reported in any group.

**TABLE 1:** Baseline characteristics.

	Cocoa (*n* = 15)	EC (*n* = 14)	MX (*n* = 15)	EC + MX (*n* = 14)	Placebo (*n* = 15)
Age (years)	32 ± 4	36 ± 5	35 ± 4	39 ± 5	28 ± 3
Sex (males/females)	3/12	3/11	4/11	5/9	1/14
Anthropometric parameters					
BMI (kg m^−2^)	24.3 ± 1.0	23.2 ± 0.9	22.2 ± 0.6	22.7 ± 0.6	21.4 ± 0.7
Body weight (kg)	69.8 ± 4.3	66.9 ± 3.1	65.8 ± 2.6	65.7 ± 2.8	62.6 ± 3.1
Waist circumference (cm)	82.1 ± 3.6	79.0 ± 2.9	79.9 ± 2.6	78.6 ± 2.1	74.7 ± 2.5
Waist‐to‐hip‐ratio	0.84 ± 0.02	0.83 ± 0.02	0.84 ± 0.02	0.84 ± 0.02	0.82 ± 0.01
Fat mass (% body weight)	32.8 ± 1.0	31.9 ± 1.5	30.1 ± 1.3	29.4 ± 1.1	31.4 ± 0.6
Office blood pressure					
Systolic blood pressure (mm Hg)	115 ± 3	117 ± 3	116 ± 3	117 ± 3	119 ± 4
Diastolic blood pressure (mm Hg)	81 ± 2	80 ± 2	77 ± 2	78 ± 2	81 ± 2
Serum lipids					
TC (mg dL^−1^)	188 ± 9	192 ± 12	185 ± 10	192 ± 12	172 ± 9
HDL‐C (mg dL^−1^)	69 ± 4	68 ± 6	67 ± 3	68 ± 6	65 ± 3
LDL‐C (mg dL^−1^)	110 ± 9	111 ± 10	107 ± 9	113 ± 10	95 ± 9
LDL‐C to HDL‐C ratio	1.7 ± 0.2	1.7 ± 0.2	1.6 ± 0.1	1.8 ± 0.2	1.5 ± 0.2
TG (mg dL^−1^)	89 ± 7	97 ± 10	86 ± 7	75 ± 4	91 ± 7

*Note*: Data are presented as means ± SEM.

Abbreviations: EC, epicatechin; HDL‐C, HDL‐cholesterol; LDL‐C, LDL‐cholesterol; LDL‐C to HDL‐C ratio, LDL‐cholesterol to HDL‐cholesterol ratio; MX, methylxanthines; TC, total cholesterol; TG, triglycerides.

### 3.3 Vascular Function

**FIGURE 2:**
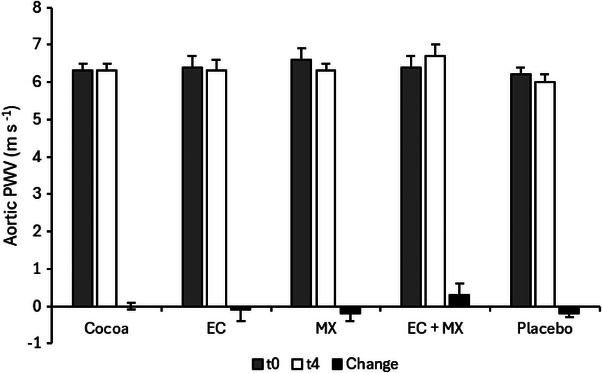
Aortic pulse wave velocity before and after the intervention and changes throughout the intervention. Data are presented as means ± SEM. Results represent aortic pulse wave velocity before (t0) and after the 4‐week intervention period (t4) and changes in each group (differences between pre‐ and posttreatment values). EC indicates epicatechin; MX, methylxanthines.

According to one‐way ANOVA, an overall treatment effect for aortic PWV was not observed (*p* = 0.410; Figure 2). Moreover, one‐way ANOVA did not indicate an overall treatment effect for all other vascular measurements (*p* ≥ 0.05; Table 2).

**TABLE 2:** Vascular parameters before (t0) and after the 4‐week (t4) intervention period.

		Cocoa (*n* = 15)^a^	EC (*n* = 14)	MX (*n* = 15)	MX + EC (*n* = 14)^a^	Placebo (*n* = 15)	*p*
Aortic PWV (m s^−1^)	t0	6.3 ± 0.2	6.4 ± 0.3	6.6 ± 0.3	6.4 ± 0.3	6.2 ± 0.2	
	t4	6.3 ± 0.2	6.3 ± 0.3	6.3 ± 0.2	6.7 ± 0.3	6.0 ± 0.2	
	Change	0.0 ± 0.1	−0.1 ± 0.3	−0.2 ± 0.2	0.3 ± 0.3	−0.2 ± 0.1	0.410^b^
Aortic SBP (mm Hg)	t0	91.1 ± 2.4	92.2 ± 3.3	93.5 ± 4.3	95.7 ± 4.6	91.0 ± 2.6	
	t4	87.6 ± 2.6	92.0 ± 3.3	89.0 ± 2.4	94.3 ± 4.2	87.9 ± 3.0	
	Change	−3.6 ± 1.2	−0.2 ± 2.7	−4.5 ± 2.5	−1.4 ± 2.1	−3.1 ± 1.2	0.561^c^
Aortic DBP (mm Hg)	t0	65.5 ± 1.9	67.2 ± 2.4	65.1 ± 3.3	66.1 ± 2.5	65.5 ± 1.9	
	t4	61.9 ± 2.7	65.3 ± 2.4	65.2 ± 2.5	65.5 ± 2.1	60.7 ± 1.8	
	Change	−3.6 ± 2.4	−1.9 ± 2.4	0.1 ± 1.6	−0.5 ± 1.6	−4.7 ± 1.5	0.352^c^
Compliance index	t0	0.61 ± 0.02	0.56 ± 0.02	0.56 ± 0.02	0.58 ± 0.02	0.56 ± 0.03	
	t4	0.62 ± 0.01	0.57 ± 0.02	0.55 ± 0.02	0.58 ± 0.03	0.57 ± 0.03	
	Change	0.0 ± 0.02	0.01 ± 0.02	−0.01 ± 0.02	0.00 ± 0.03	0.01 ± 0.03	0.963^c^
PRT^.^ (ms)	t0	97 ± 3	100 ± 4	98 ± 3	100 ± 2	100 ± 3	
	t4	101 ± 3	103 ± 4	97 ± 2	101 ± 2	99 ± 3	
	Change	4 ± 2	4 ± 2	−1 ± 1	1 ± 2	−1 ± 2	0.087^c^
Resistance index	t0	19.0 ± 2.3	22.0 ± 3.5	19.4 ± 2.1	17.7 ± 1.8	19.0 ± 3.1	
	t4	16.4 ± 1.8	19.3 ± 2.4	18.4 ± 1.4	16.6 ± 1.0	18.1 ± 1.7	
	Change	−2.6 ± 1.5	−2.6 ± 1.9	−0.9 ± 1.9	−1.1 ± 1.7	−0.9 ± 2.3	0.918^c^
AIX (%)	t0	1.7 ± 3.3	2.9 ± 4.2	2.4 ± 5.3	8.2 ± 6.8	0.5 ± 4.2	
	t4	1.1 ± 3.1	2.4 ± 5.4	−1.5 ± 4.4	7.9 ± 6.7	−2.9 ± 4.3	
	Change	−0.6 ± 1.5	−0.5 ± 2.5	−3.9 ± 2.0	−0.3 ± 3.7	−3.4 ± 2.3	0.873^b^
AIX 75 (%)	t0	−4.5 ± 2.8	−3.5 ± 3.7	−3.3 ± 5.5	−0.3 ± 6.4	−6.3 ± 3.8	
	t4	−5.6 ± ‐5.6	−4.8 ± 5.2	−7.3 ± 4.3	−0.6 ± 6.2	−9.8 ± 4.0	
	Change	−1.1 ± 1.6	−1.2 ± 2.5	−4.0 ± 2.1	−0.3 ± 4.0	−3.5 ± 2.2	0.967^b^
Brachial PWV right^.^(m s^−1^)	t0	8.8 ± 0.2	8.9 ± 0.2	9.0 ± 0.3	8.7 ± 0.3	8.7 ± 0.2	
	t4	8.5 ± 0.2	9.0 ± 0.2	9.1 ± 0.3	8.7 ± 0.2	8.5 ± 0.1	
	Change	−0.3 ± 0.1	0.1 ± 0.1	0.1 ± 0.2	0.0 ± 0.1	−0.2 ± 0.2	0.146^c^
Brachial PWV left^.^(m s^−1^)	t0	8.7 ± 0.1	9.1 ± 0.2	9.1 ± 0.2	9.0 ± 0.2	8.9 ± 0.3	
	t4	8.5 ± 0.2	9.0 ± 0.2	8.8 ± 0.2	8.9 ± 0.2	8.6 ± 0.2	
	Change	−0.2 ± 0.1	−0.1 ± 0.1	−0.3 ± 0.2	−0.1 ± 0.1	−0.3 ± 0.1	0.554^b^
ABI right	t0	1.27 ± 0.03	1.29 ± 0.03	1.29 ± 0.03	1.26 ± 0.02	1.27 ± 0.02	
	t4	1.30 ± 0.02	1.28 ± 0.02	1.32 ± 0.02	1.25 ± 0.03	1.28 ± 0.02	
	Change	0.03 ± 0.02	−0.01 ± 0.02	0.03 ± 0.02	−0.01 ± 0.02	0.01 ± 0.02	0.460^b^
ABI left	t0	1.27 ± 0.03	1.28 ± 0.02	1.30 ± 0.03	1.26 ± 0.02	1.26 ± 0.02	
	t4	1.29 ± 0.02	1.29 ± 0.02	1.31 ± 0.02	1.25 ± 0.02	1.27 ± 0.02	
	Change	0.02 ± 0.02	0.00 ± 0.02	0.01 ± 0.02	−0.01 ± 0.02	0.01 ± 0.02	0.906^c^
Office SBP (mm Hg)	t0	115.1 ± 2.7	117.0 ± 3.0	116.4 ± 3.3	117.3 ± 3.0	119.1 ± 3.8	
	t4	115.3 ± 2.2	117.1 ± 2.6	115.3 ± 2.3	117.6 ± 2.9	116.6 ± 3.2	
	Change	0.1 ± 1.7	0.1 ± 1.6	−1.1 ± 1.5	0.4 ± 3.6	−2.5 ± 1.3	0.915^b^
Office DBP (mm Hg)	t0	81.2 ± 2.0	79.6 ± 1.5	76.8 ± 2.3	77.6 ± 2.1	80.8 ± 2.1	
	t4	78.1 ± 2.0	79.9 ± 2.0	77.5 ± 1.5	77.9 ± 1.8	77.2 ± 1.4	
	Change	−3.1 ± 1.5	0.3 ± 1.8	0.7 ± 1.3	0.4 ± 2.0	−3.6 ± 1.3	0.216^b^
Endothelin‐1 (pg mL^−1^)	t0	1.67 ± 0.17	2.03 ± 0.26	1.89 ± 0.15	1.96 ± 0.13	2.21 ± 0.28	
	t4	1.89 ± 0.15	1.90 ± 0.22	1.77 ± 0.17	1.64 ± 0.14	2.22 ± 0.34	
	Change	0.22 ± 0.11	−0.13 ± 0.10	−0.12 ± 0.21	−0.31 ± 0.20	0.01 ± 0.15	0.154^b^

*Note*: Data are presented as means ± SEM.

Abbreviations: ABI, ankle brachial index; AIX, augmentation index; AIX75, augmentation index standardized for 75 beats per minute; DBP, diastolic blood pressure; EC, epicatechin; MX, methylxanthines; PRT, radial pulse raise time; PWV, pulse wave velocity; SBP, systolic blood pressure.

^a^ The values for one subject were missing for parameters derived from aortic PWV (aortic SBP, aortic DBP, compliance index, resistance index, PRT AIX, AIX 75).

^b^ According to the Kruskal–Wallis test.

^c^ According to one‐way analysis of variance (one‐way ANOVA).

### 3.4 Serum Lipids

The results on serum lipids are presented in Table 3. According to Kruskal–Wallis test, an overall treatment effect for TC, LDL‐C, HDL‐C, LDL‐C to HDL‐C ratio, and TG, based on the differences between pre‐ and posttreatment values, could not be found for group‐specific variations at the 5% type I error level (*p *≥ 0.05 for each parameter).

**TABLE 3:** Serum lipids before (t0) and after the 4‐week intervention period (t4).

		Cocoa (*n* = 15)	EC (*n* = 14)	MX (*n* = 15)	EC + MX (*n* = 14)	Placebo (*n* = 15)	*p*
TC (mg dL^−1^)	t0	188 ± 9	192 ± 12	185 ± 10	192 ± 12	172 ± 9	
	t4	193 ± 10	181 ± 13	179 ± 10	195 ± 11	167 ± 10	
	Change	5 ± 3	−12 ± 9	−6 ± 4	3 ± 4	−4 ± 4	0.228^a^
LDL‐C (mg dL^−1^)	t0	110 ± 9	111 ± 10	107 ± 9	113 ± 10	95 ± 9	
	t4	113 ± 9	101 ± 9	103 ± 9	108 ± 9	91 ± 9	
	Change	3 ± 3	−10 ± 7	−4 ± 3	−5 ± 4	−4 ± 3	0.281^a^
HDL‐C (mg dL^−1^)	t0	69 ± 4	68 ± 6	67 ± 3	68 ± 6	65 ± 3	
	t4	68 ± 3	67 ± 6	66 ± 3	73 ± 6	63 ± 3	
	Change	−1 ± 2	−1 ± 2	0 ± 1	5 ± 2	−3 ± 2	0.068^a^
LDL‐C/HDL‐C	t0	1.7 ± 0.2	1.7 ± 0.2	1.6 ± 0.1	1.8 ± 0.2	1.5 ± 0.2	
	t4	1.7 ± 0.2	1.6 ± 0.2	1.6 ± 0.1	1.6 ± 0.2	1.5 ± 0.2	
	Change	0.0 ± 0.1	−0.2 ± 0.1	0.0 ± 0.1	−0.2 ± 0.1	0.0 ± 0.0	0.262^a^
TG (mg dL^−1^)	t0	89 ± 7	97 ± 10	86 ± 7	75 ± 4	91 ± 7	
	t4	90 ± 7	94 ± 9	93 ± 11	77 ± 6	86 ± 9	
	Change	1 ± 3	−4 ± 8	7 ± 9	3 ± 6	−6 ± 6	0.672^a^

*Note*: Data are presented as means ± SEM.

Abbreviations: EC, epicatechin; HDL‐C, HDL‐cholesterol; LDL‐C, LDL‐cholesterol; MX, methylxanthines; TC, total cholesterol; TG, triglycerides.

^a^ According to the Kruskal–Wallis test.

## 4 Discussion

To the best of our knowledge, this is the first RCT to compare the effects of full flavanol‐rich cocoa powder with those of pure cocoa ingredients (EC, MX, or a mixture of EC and MX) on vascular parameters and serum lipid levels. In contrast to our expectations, no changes in aortic PWV or any other outcome measures were observed after 4 weeks of supplementation with either cocoa powder or pure cocoa ingredients. Thus, the goals of our systematically planned intervention study to gain further insights into the cardiometabolic mechanisms of longer‐term cocoa intake could not be reached.

Several reasons for these unexpected findings can be excluded. The amount of CF ingested through cocoa in our study was similar to that reported in an earlier RCT [7]; thus, the amount of active ingredients administered in this study was not too low. As requested, our participants renounced consumption of cocoa products and reduced their food intake of cocoa ingredients from the run‐in phase up to the end of the intervention period. Except for caffeine, the intake of other cocoa ingredients (EC, flavan‐3‐ols, and TB) by food revealed only a minor share of the total ingredient intake during the intervention (Table S3). Regarding caffeine intake, bias is unlikely because a recently published study by Ottaviani et al. [37] showed that the consumption of a dexanthinated flavan‐3‐ol‐rich cocoa drink enriched with 112 mg caffeine did not modulate EC bioavailability in healthy adults in contrast to the increase observed when a drink enriched with 94 mg TB and 11 mg caffeine is consumed [37]. Thus, masking of supplementation effects is rather unlikely. For power calculation, we used recently published data demonstrating an average decrease in PWV of at least 1.0 m s^−1^ after cocoa supplementation in healthy young adults compared with controls [8]. At least for full cocoa powder, our study was sufficiently powered to reach the study goals.

It can be, however, speculated that variations in the composition of cocoa products investigated and methodological variations may lead to false negative or false positive results. In an RCT with a parallel group design involving healthy adults, Rodriguez‐Mateos et al. supplemented encapsulated cocoa extracts containing a higher amount of total flavan‐3‐ols (690 mg DP 1–10 including 130 mg EC) compared to the cocoa powder used in this study (≥ 200 mg DP 1–10, 37 mg EC) but less MX (80 mg TB, 20 mg caffeine). The participants were instructed to consume the capsules with their breakfast [8]. In their crossover trial, Grassi et al. [7] offered daily water‐based cocoa drinks prepared from cocoa with similar amounts of CF, EC, TB, and caffeine (product of the same manufacturer as used in this study) to be consumed at a fasting state (3 h after breakfast). Since an effective luminal absorption of cocoa ingredients, such as monomeric flavan‐3‐ols (EC) and MX, may be influenced by other nutrients, the timing of cocoa consumption (postprandial or with a meal) might be decisive for its physiological effects. The coincident consumption of TB and caffeine with CF has shown to increase the bioavailability of EC (AUC_0‐4 h_ +22% ± 5% of structurally related EC metabolites) [15]. A recently published trial by Ottaviani et al. [37] involving 10 healthy adults on the pharmacokinetics of structurally related EC metabolites in plasma after consumption of a CF‐rich drink found higher AUC values (increase in AUC_0‐6 h_ only by +9% ± 3%) when the drink was enriched with MX (94 mg TB, 11 mg caffeine), but not by enrichment with caffeine (112 mg) [30]. Probably, other food ingredients may alter flavan‐3‐ol absorption processes. Moreover, EC bioavailability largely differs between individuals. A parallel group design, which is more sensitive to interindividual variabilities, may partly explain the lack of effects in the study groups.

Rodriguez‐Mateos et al. [8] and Grassi et al. [7] applied a tonometric procedure (SphygmoCor system) to record pulse waves between the femoral and carotid arteries. Although the VascAssist 2 methodology has been validated against the tonometry pulse waves measured using SphygmoCor [23], methodological differences cannot be completely excluded.

From a physiological perspective, all participants exhibited almost optimal lipid profiles and BP values in the lower normal range (Table 3). Therefore, the potential for improvements of vascular functions and serum lipid profiles by cocoa powder or pure cocoa ingredients was marginal. A generally accepted prerequisite to evaluate potential mechanisms of food and food ingredients at the physiological level is to conduct studies on healthy subjects; thus, we chose a group of younger, obviously healthy adults for our trial.

An essential strength of this intervention study is its double‐blind, placebo‐controlled design. Compliance with restrictions on diet and lifestyle was controlled by obtaining food records and assessing physical activity, respectively. Therefore, any bias related to the results by changes in diet and lifestyle is rather unlikely. Furthermore, hormonal changes throughout the menstrual cycle may affect BP [38] and serum lipid levels [39]. Since the present study lasted 4 weeks and premenopausal women were always studied at the onset of their menstrual cycle, any bias due to the actual hormonal situation on BP and serum lipids is unlikely.

This study has some limitations. First, CF‐derived metabolites formed after absorption in the small intestine and conjugated by human phase II enzymes (structurally related EC metabolites) and metabolites generated by gut microbiota, subsequently absorbed and conjugated by human enzymes (ring‐fission metabolites), all potential mediators of metabolic cocoa effects, have not been investigated. Furthermore, interindividual differences in the bioavailability of flavan‐3‐ols may have hidden potential protective effects derived from the supplementation. Thus, CF uptake, which may be relevant to cardiometabolic effects, remains unclear. Second, the trial had a parallel group design in which the interindividual variability was much higher than that in a crossover study. Third, the sex ratio was unbalanced; stratification according to sex will be useful in future studies to exclude potential bias by sex. Fourth, the sample size calculation only considered the treatment effect of flavanol‐rich cocoa on PWV because the corresponding data for the other treatments were unavailable; therefore, we cannot rule out the possibility that the study is underpowered for the EC, MX, and EC + MX treatment arms.

In conclusion, neither daily intake of flavanol‐rich cocoa powder nor ingestion of pure cocoa ingredients influenced the selected outcome variables (vascular functions and serum lipid profiles) of healthy young nonsmoking adults; thus, we could not verify the effects of EC and MX as potential mediators of cocoa effects on cardiovascular health. Consequently, RCTs involving subjects with increased cardiovascular risk (e.g., increased BP and impaired serum lipid profiles) are necessary to clarify the contribution of various cocoa ingredients on markers of cardiometabolic health. To understand the effects of specific CF metabolites (structurally related and ring‐fission metabolites) and of the interindividual variability in flavan‐3‐ol bioavailability, their excretion in 24‐h urine should also be determined.

## Conflicts of Interest

The authors declare no conflicts of interest.

## Supporting information



Supporting Information

## Data Availability

Data described in the manuscript will not be made available as the participants were assured in the informed consent form that personal data will not be disclosed to third parties.
