# Supplementary material for: Cardiometabolic Impact of Encapsulated Cocoa Powder and Pure Cocoa Ingredients Supplementation: A Comparative Placebo‐Controlled RCT in Adults
Source: Mol Nutr Food Res. 2025 Feb 3;69(5):e202400490. doi: 10.1002/mnfr.202400490 (PMC11874242; doi:10.1002/mnfr.202400490)
Supplement: Supplementary file 1 — Supporting Information [file MNFR-69-e202400490-s001.docx]

**Supporting Information**

**“Cardiometabolic impact of encapsulated cocoa powder and pure cocoa ingredients supplementation – a comparative placebo-controlled RCT in adults”**

Janina Weigant, Anuschka Afchar, Meike Barzen, Lisa Dicks, Benno F. Zimmermann, Matthias Schmid, Leonie Weinhold, Birgit Stoffel-Wagner, Jörg Ellinger, Peter Stehle, Sabine Ellinger

Corresponding author: Sabine Ellinger, Institute of Nutritional and Food Sciences, Human Nutrition, University of Bonn, Bonn, Germany. E-mail: [ellinger@uni-bonn.de](mailto:ellinger@uni-bonn.de)

**Table S1.** Composition of cocoa powder used for preparing verum capsules.

|  | Content of a daily serving size |
| --- | --- |
| Energy, kcal | 8.5 |
| Macronutrients |  |
| Protein, g | 0.6 |
| Fat, g | 0.3 |
| Carbohydrates, g | 0.6 |
| Flavan-3-ols |  |
| Epicatechin, mg | 37.3 |
| Total flavan-3-ols (∑ DP1-10), mg | ≥ 200 |
| Methylxanthines |  |
| Caffeine, mg | 25^a)^ |
| Theobromine, mg | 329^b)^ |

DP, degree of polymerization. Data on the content of energy, macronutrients and total flavan-3-ols refer to 2.5 g ACTICOA cocoa powder and were provided by the manufacturer; own data on epicatechin, caffeine and theobromine obtained by analysis with HPLC. ^a)^ 8.3 mg from ACTICOA cocoa powder enriched with 16.7 mg pure caffeine, ^b)^ 57 mg from ACTICOA cocoa powder enriched with 272 mg pure theobromine.

**Table S2.** Ingredients of the capsules used for the different interventions.

|  | Cocoa | EC | MX | EC + MX | Placebo |
| --- | --- | --- | --- | --- | --- |
| Flavan-3-ols |  |  |  |  |  |
| Epicatechin, mg | 37.3 | 37.3 | 0 | 37.3 | 0 |
| Total flavan-3-ols (∑ DP1-10), mg | ≥ 200 | − | 0 | − | 0 |
| Methylxanthines |  |  |  |  |  |
| Theobromine, mg | 329 | 0 | 329 | 329 | 0 |
| Caffeine, mg | 25 | 0 | 25 | 25 | 0 |

DP, degree of polymerization; EC, epicatechin; MX, methylxanthines.

**Table S3.** Anthropometric parameters, dietary intake^1^ and physical activity^1^ at the first visit (t0) and at the second visit 4-weeks later (t4).

|  | Cocoa  (*n* = 15) | | | | | | EC  (*n* = 15) | | | | | | MX  (*n* = 14) | | | | | | EC + MX  (*n* = 15) | | | | | | Placebo  (*n* = 14) | | | | | | |
| --- | --- | --- | --- | --- | --- | --- | --- | --- | --- | --- | --- | --- | --- | --- | --- | --- | --- | --- | --- | --- | --- | --- | --- | --- | --- | --- | --- | --- | --- | --- | --- |
|  | t0 | | | t4 | | | t0 | | | t4 | | | t0 | | | t4 | | | t0 | | | t4 | | | t0 | | | t4 | | | |
| Anthropometric parameters |  |  |  |  |  |  |  |  |  |  |  |  |  |  |  |  |  |  |  |  |  |  |  |  |  |  |  |  |  |  |  |
| BW (kg) | 69.8 | ± | 4.3 | 69.8 | ± | 4.3 | 66.9 | ± | 3.1 | 66.6 | ± | 3.1 | 65.9 | ± | 2.6 | 65.9 | ± | 2.6 | 65.7 | ± | 2.8 | 65.7 | ± | 2.8 | 62.6 | ± | 3.1 | 62.7 | ± | 3.0 |  |
| FM (% BW) | 32.8 | ± | 1.0 | 32.5 | ± | 1.1 | 31.9 | ± | 1.5 | 32.2 | ± | 1.5 | 30.1 | ± | 1.3 | 29.8 | ± | 1.3 | 29.4 | ± | 1.1 | 30.4 | ± | 1.5 | 31.4 | ± | 0.6 | 31.2 | ± | 0.6 |  |
| WC (cm) | 82.1 | ± | 3.6 | 81.1 | ± | 3.5 | 79.0 | ± | 2.9 | 79.6 | ± | 2.9 | 79.9 | ± | 2.6 | 78.6 | ± | 2.7 | 78.6 | ± | 2.1 | 78.0 | ± | 2.0 | 74.7 | ± | 2.5 | 74.8 | ± | 2.5 |  |
| WHR | 0.84 | ± | 0.02 | 0.84 | ± | 0.03 | 0.83 | ± | 0.02 | 0.83 | ± | 0.02 | 0.84 | ± | 0.02 | 0.83 | ± | 0.02 | 0.84 | ± | 0.02 | 0.83 | ± | 0.02 | 0.82 | ± | 0.01 | 0.82 | ± | 0.02 |  |
| Dietary intake |  |  |  |  |  |  |  |  |  |  |  |  |  |  |  |  |  |  |  |  |  |  |  |  |  |  |  |  |  |  |  |
| Energy (kcal) | 1782 | ± | 140 | 1747 | ± | 132 | 1593 | ± | 154 | 1813 | ± | 139 | 1721 | ± | 101 | 1844 | ± | 141 | 2171 | ± | 187 | 2068 | ± | 154 | 1737 | ± | 100 | 1618 | ± | 91 |  |
| Protein (g) | 67 | ± | 7 | 68 | ± | 6 | 60 | ± | 6 | 62 | ± | 5 | 65 | ± | 5 | 63 | ± | 6 | 82 | ± | 8 | 75 | ± | 7 | 65 | ± | 4 | 60 | ± | 3 |  |
| Fat (g) | 78 | ± | 8 | 76 | ± | 8 | 66 | ± | 7 | 86 | ± | 9 | 78 | ± | 8 | 74 | ± | 9 | 97 | ± | 14 | 90 | ± | 9 | 71 | ± | 7 | 64 | ± | 5 |  |
| SFA (g) | 28 | ± | 4 | 29 | ± | 4 | 26 | ± | 3 | 31 | ± | 4 | 28 | ± | 3 | 26 | ± | 4 | 34 | ± | 4 | 34 | ± | 5 | 24 | ± | 2 | 25 | ± | 3 |  |
| MUFA (g) | 29 | ± | 3 | 26 | ± | 3 | 21 | ± | 2 | 29 | ± | 3 | 28 | ± | 3 | 27 | ± | 4 | 36 | ± | 8 | 32 | ± | 3 | 24 | ± | 3 | 21 | ± | 2 |  |
| PUFA (g) | 15 | ± | 2 | 15 | ± | 2 | 14 | ± | 2 | 19 | ± | 3 | 17 | ± | 1 | 16 | ± | 2 | 20 | ± | 3 | 17 | ± | 2 | 16 | ± | 3.1 | 13.0 | ± | 1.5 |  |
| Chol (mg) | 253 | ± | 63 | 224 | ± | 55 | 222 | ± | 41 | 272 | ± | 52 | 242 | ± | 41 | 241 | ± | 65 | 288 | ± | 61 | 277 | ± | 63 | 243 | ± | 48 | 242 | ± | 46 |  |
| CHO (g) | 207 | ± | 17 | 206 | ± | 16 | 193 | ± | 21 | 199 | ± | 15 | 197 | ± | 11 | 242 | ± | 14 | 253 | ± | 20 | 250 | ± | 18 | 220 | ± | 10 | 204 | ± | 10 |  |
| Fiber (g) | 25 | ± | 3 | 28 | ± | 2 | 28 | ± | 3 | 25 | ± | 2 | 30 | ± | 4 | 29 | ± | 4 | 26 | ± | 4 | 26 | ± | 3 | 24 | ± | 3 | 26 | ± | 3 |  |
| Epicatechin (mg) | 4.3 | ± | 3.0 | 3.8 | ± | 2.8 | 0.9 | ± | 0.6 | 2.4 | ± | 0.7 | 2.2 | ± | 0.50 | 1.8 | ± | 0.4 | 1.4 | ± | 0.5 | 1.1 | ± | 0.5 | 1.4 | ± | 0.6 | 1.1 | ± | 0.4 |  |
| Total flavan-3-ols^a)^ (mg) | 27.0 | ± | 17.2 | 26.3 | ± | 20.3 | 7.0 | ± | 5.8 | 23.8 | ± | 8.1 | 15.5 | ± | 3.7 | 15.4 | ± | 4.5 | 12.4 | ± | 4.9 | 6.3 | ± | 2.7 | 11.1 | ± | 4.8 | 7.7 | ± | 2.9 |  |
| Theobromine (mg) | 0.00 | ± | 0.00 | 0.03 | ± | 0.03 | 0.04 | ± | 0.04 | 0.04 | ± | 0.04 | 0.40 | ± | 0.21 | 0.37 | ± | 0.17 | 0.21 | ± | 0.15 | 0.29 | ± | 0.21 | 0.13 | ± | 0.09 | 0.17 | ± | 0.09 |  |
| Caffeine (mg) | 56 | ± | 10 | 48 | ± | 11 | 57 | ± | 10 | 55 | ± | 10 | 46 | ± | 11 | 55 | ± | 14 | 55 | ± | 9 | 44 | ± | 10 | 21 | ± | 6 | 19 | ± | 5 |  |
| Physical activity |  |  |  |  |  |  |  |  |  |  |  |  |  |  |  |  |  |  |  |  |  |  |  |  |  |  |  |  |  |  |  |
| Steps | 7800 | ± | 894 | 7654 | ± | 823 | 6837 | ± | 1001 | 7689 | ± | 549 | 8606 | ± | 566 | 9597 | ± | 1028 | 11973 | ± | 912 | 10755 | ± | 1287 | 8469 | ± | 803 | 8349 | ± | 782 |  |
| Data are presented as means ± SEM. ^1^data were calculated as mean value per day based on investigations performed three days before each visit. ^a)^ Determined as sum of (−)-epicatchin, (+)-catechin and procyandins (dimers – decamers). BW, body weight; CHO, carbohydrates; Chol, cholesterol; EC, epicatechin; FM, fat mass; MX, methylxanthines; SFA, saturated fatty acids; WC, waist circumference; WHR, waist-to-hip-ratio. | | | | | | | | | | | | | | | | | | | | | | | | | | | | | | | |
